# Whole Exome Sequencing Identifies *APCDD1* and *HDAC5* Genes as Potentially Cancer Predisposing in Familial Colorectal Cancer

**DOI:** 10.3390/ijms22041837

**Published:** 2021-02-12

**Authors:** Diamanto Skopelitou, Beiping Miao, Aayushi Srivastava, Abhishek Kumar, Magdalena Kuswick, Dagmara Dymerska, Nagarajan Paramasivam, Matthias Schlesner, Jan Lubinski, Kari Hemminki, Asta Försti, Obul Reddy Bandapalli

**Affiliations:** 1 Molecular Genetic Epidemiology, German Cancer Research Center (DKFZ), 69120 Heidelberg, Germany; mando.skopelitou@yahoo.de (D.S.); b.miao@kitz-heidelberg.de (B.M.); srivastava.aayhushi97@gmail.com (A.S.); abhishek@ibioinformatics.org (A.K.); k.hemminki@dkfz.de (K.H.); a.foersti@kitz-heidelberg.de (A.F.); 2 Hopp Children’s Cancer Center (KiTZ), 69120 Heidelberg, Germany; 3 Division of Pediatric Neurooncology, German Cancer Research Center (DKFZ) and German Cancer Consortium (DKTK), 69120 Heidelberg, Germany; 4 Medical Faculty, Heidelberg University, 69120 Heidelberg, Germany; 5 Institute of Bioinformatics, International Technology Park, Bangalore 560066, India; 6 Manipal Academy of Higher Education (MAHE), Manipal 576104, India; 7 Department of Genetics and Pathology, Pomeranian Medical University, 71252 Szczecin, Poland; magdalenakuswik@gmail.com (M.K.); dymerska@pum.edu.pl (D.D.); lubinski@pum.edu.pl (J.L.); 8 Computational Oncology, Molecular Diagnostics Program, National Center for Tumor Diseases (NCT), 69120 Heidelberg, Germany; n.paramasivam@dkfz.de; 9 Bioinformatics and Omics Data Analytics, German Cancer Research Center (DKFZ), 69120 Heidelberg, Germany; m.schlesner@dkfz.de; 10 Cancer Epidemiology, German Cancer Research Center (DKFZ), 69120 Heidelberg, Germany; 11 Biomedical Center, Faculty of Medicine in Pilsen, Charles University in Prague, 30605 Pilsen, Czech Republic

**Keywords:** *APCDD1*, *HDAC5*, 5´UTR, germline variant, familial colorectal cancer, whole exome sequencing, promoter activity

## Abstract

Germline mutations in predisposition genes account for only 20% of all familial colorectal cancers (CRC) and the remaining genetic burden may be due to rare high- to moderate-penetrance germline variants that are not explored. With the aim of identifying such potential cancer-predisposing variants, we performed whole exome sequencing on three CRC cases and three unaffected members of a Polish family and identified two novel heterozygous variants: a coding variant in APC downregulated 1 gene (*APCDD1*, p.R299H) and a non-coding variant in the 5′ untranslated region (UTR) of histone deacetylase 5 gene (*HDAC5*). Sanger sequencing confirmed the variants segregating with the disease and Taqman assays revealed 8 additional *APCDD1* variants in a cohort of 1705 familial CRC patients and no further *HDAC5* variants. Proliferation assays indicated an insignificant proliferative impact for the *APCDD1* variant. Luciferase reporter assays using the *HDAC5* variant resulted in an enhanced promoter activity. Targeting of transcription factor binding sites of SNAI-2 and TCF4 interrupted by the *HDAC5* variant showed a significant impact of TCF4 on promoter activity of mutated *HDAC5*. Our findings contribute not only to the identification of unrecognized genetic causes of familial CRC but also underline the importance of 5’UTR variants affecting transcriptional regulation and the pathogenesis of complex disorders.

## 1. Introduction

Whole exome sequencing (WES) is gaining relevance for molecular genetic research of familial cancer and the identification of new cancer-predisposing variants. Among hereditary malignancies, colorectal cancer (CRC) shows one of the highest proportions of familial cases and heritable factors have been estimated to account for about 35% of CRC risk, according to twin studies [1]. Cancer-predisposing germline mutations of *APC, MUTYH* and mismatch repair genes are already known to be associated with familial CRC and to lead to: phenotypes of well-defined Mendelian CRC syndromes, familial adenomatous polyposis (FAP), resulting from *APC* gene mutations; *MUTYH*-associated polyposis (MAP); and Lynch syndrome, a hereditary non-polyposis colon cancer (HNPCC) syndrome caused by mismatch repair gene mutations (*MLH1, MSH2, MSH6, PMS2* and *EPCAM*) [2]. CRC fulfilling the diagnostic criteria of Lynch syndrome but not linked to pathogenic mismatch repair gene mutations or the resulting microsatellite instability has been classified as Familial Colorectal Cancer Type X (FCCTX). Although FCCTX is considered as a heterogeneous group with unknown genetic etiology, candidate genes such as *CENPE, KIF24, GALNT12, ZNF367, GABBR2* and *BMP4* have been suggested as cancer predisposing in these patients [3]. Sequencing studies have further identified germline variants in *HNRNPA0* and *WIF1* genes in a family with susceptibility to multiple early onset cancers including CRC [4] as well as a germline mutation in *NTHL1* gene in three unrelated families with adenomatous polyposis and various cancer types including CRC [5,6]. Moreover, germline as well as somatic mutations in *POLE* and *POLD1* genes have been associated with both sporadic and familial CRC contributing to the genetic understanding of CRC inheritance [7,8]. Nevertheless, germline mutations in these or other established predisposition genes account for only 5 to 10% of all CRC [9]. Since the genetic background of most familial CRC cases has still not been sufficiently explored, the application of WES on these patients within pedigree-based studies bears great potential for the exploration of the remaining genetic burden.

As 98% of the genome is non-coding, a high proportion of variants are identified in this region [10] and are gaining relevance in the understanding of inherited cancer predisposition [11,12]. The great potential impact of variants within the 5′ untranslated region (UTR) of a gene or up to 1 kb upstream of transcription start sites can be attributed to possible changes in transcriptional regulatory elements, such as binding motifs in promoters, enhancers or super-enhancers. On the other hand, the 3′UTR of a gene and the flanking region downstream of transcription end sites can carry essential miRNA binding sites where small RNAs containing the respective complementary sequence can post-transcriptionally attach; hence, mRNA translation can be suppressed leading to the inhibition of gene expression [13]. By this and many other means, non-coding regions can play an important role in transcriptional and post-transcriptional regulation of gene expression, which is why genetic variation of non-coding DNA has to be considered in the analysis and prioritization of potentially cancer-causing variants.

We have developed the Familial Cancer Variant Prioritization Pipeline (FCVPPv2) for evaluation of both coding and non-coding variants and implemented it in the prioritization of novel missense variants in the tumor suppressor genes *DICER1* in Hodgkin lymphoma and *CPXM1* in papillary thyroid cancer and in the pathways enriched in these entities [14,15,16,17]. In the present study, tools such as the Combined Annotation Dependent Depletion v1.4 (CADD) tool [18], SNPnexus [19] and the Bedtools intersect function were applied as part of the non-coding analysis of our pipeline in order to identify important regulatory elements. Using FCVPPv2 and literature mining, we were able to prioritize two novel heterozygous variants in a family affected by CRC, a coding variant in the APC downregulated 1 (*APCDD1*) gene and a non-coding variant in the histone deacetylase 5 (*HDAC5*) gene. Whereas the *APCDD1* variant was identified in 8 additional cases among 1705 CRC families, cell proliferation assays indicated an insignificant proliferative impact for the variant. We did not find any other familial CRC cases with the *HDAC5* variant, but functional experiments showed a significant impact of the 5′UTR variant on expression of *HDAC5*, involved in cellular processes such as proliferation, differentiation, apoptosis and cell cycle progression. Luciferase reporter assays resulted in enhanced promoter activity of the *HDAC5* gene carrying this variant compared to the wild-type sequence and targeting of transcription factor binding sites interrupted by this variant showed an impact of TCF4 on promoter activity of mutated *HDAC5*.

## 2. Results

### 2.1. FCVPPv2 Analysis of Coding Variants Prioritized a Missense Variant in APCDD1 Gene

**Figure 1:**
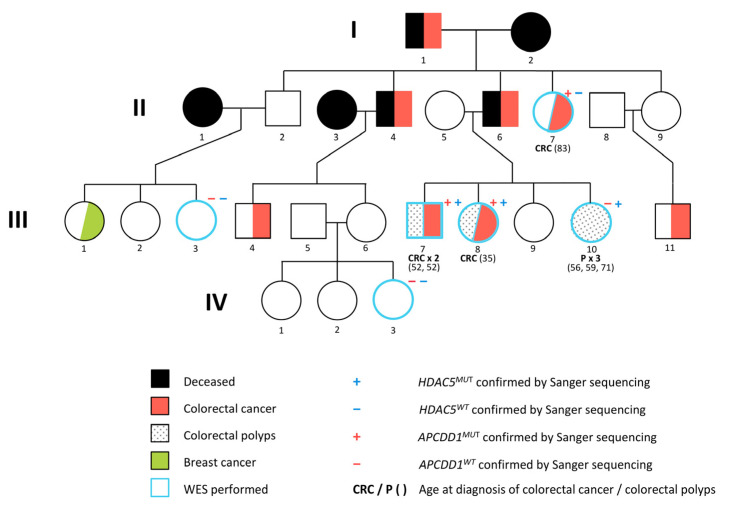
Pedigree of the CRC (colorectal cancer) family with *APCDD1* and *HADC5* variants analyzed in this study.

Application of WES on the studied CRC-affected family identified 13,733 variants with MAF (minor allele frequency) ≤ 0.1%. Filtering according to the probability for each family member of being a Mendelian case (Figure 1, Supplementary Table S1) narrowed down the number of identified variants to 783. For analysis of coding variants (*n* = 101), synonymous variants (*n* = 35) were filtered out as they are generally considered to play a minor role in the development of diseases and cancer. The remaining 66 nonsynonymous variants, frameshift deletions/insertions or variants of unknown significance were further evaluated. Application of the PHRED-like CADD score cut-off of ≥10 reduced the number of variants to 51 and screening according to the three conservational scores GERP, PhastCons and PhyloP further narrowed down the number to 38 variants. Eighteen variants were annotated and at least 3 out of 4 intolerance scores were favorable and were further considered for deleteriousness screening. Since 12 of the variants did not fulfill the criterion of being annotated as deleterious by at least 60% of all deleteriousness scores, they were excluded. Lastly, 7 nonsynonymous variants passed all the criteria considering a MAF of 0.1% in the non-Finnish European population of gnomAD database: *APCDD1* (p.R299H), *FLNC* (p.G553S), *KCNH6* (p.L403V), *LSR* (p.A139D), MTX1 (p.Y228C), *SDS* (p.T185I), *ZW10* (p.A732P) (Table 1).

SNAP^2^ analysis indicated a functional effect of the amino acid substitutions induced by the variants in the *APCDD1* (p.R299H), *ZW10* (p.A732P) and *LSR* (p.A139D) genes by predicting scores of >50, whereas application of CGI did not identify any cancer drivers among the variants. The lipolysis-stimulated lipoprotein receptor encoded by the *LSR* gene is generally known to play a role in metabolism by inducing the uptake of triglyceride-rich lipoproteins like chylomicrons, LDL and VLDL from blood into cells. Since further literature mining did not reveal any association with colorectal carcinogenesis, the identified variant in the *LSR* gene was considered to be of minor impact on the development of CRC in the studied family. The *ZW10* gene encodes a protein of the mitotic checkpoint controlling chromosome segregation during cell division. On the background of causing chromosomal instability when mutated in a model system, Wang et al. have identified two somatic missense variants in *ZW10* gene (p.N123T, p.S623G) in a panel of CRCs [21]. As the prioritized *ZW10* variant identified in the studied family (p.A732P) is not located in the adjacent regions and is moreover located close to the end of the protein (779aa), its functional and potentially cancer-predisposing impact was considered as improbable.

**Figure 2:**
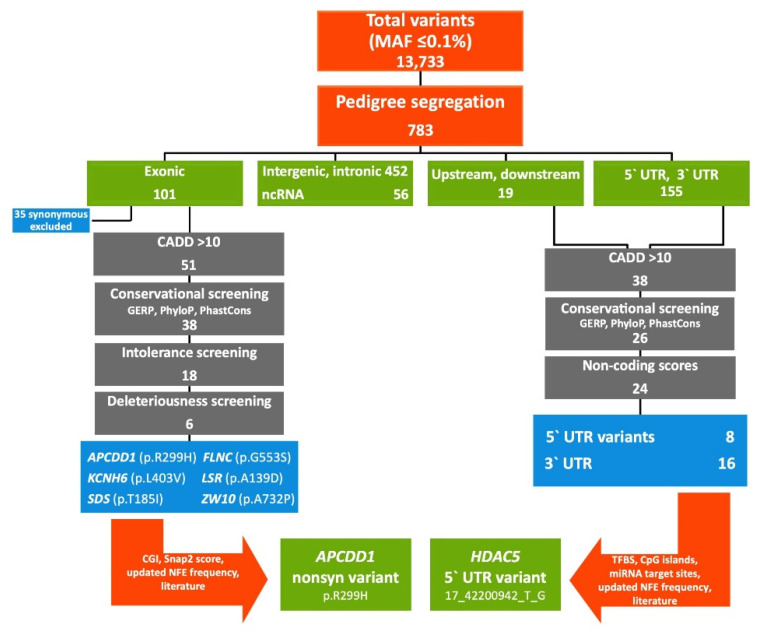
Graphical representation of the filtering process according to the Familial Cancer Variant Prioritization Pipeline version 2 (FCVPPv2).

The APCDD1 variant is located in the second of two functional APCDDC domains (51–283, 284–490), according to Interpro, Pfam and SMART [22,23,24], required for interaction with Wnt ligands and their receptors. Since the APCDD1 gene has been linked to CRC by being involved in the Wnt signaling pathway as a direct target of the beta-Catenin/TCF4 complex, the associated variant (p.R299H) was prioritized as the top cancer-predisposing candidate of all identified missense variants (Figure 2) [25]. Pedigree segregation of the APCDD1 variant was checked by IGV (Integrative Genomics Viewer) and further confirmed by targeted Sanger sequencing showing the heterozygous variant (p.R299H) for the two CRC cases (III7, III8) and the individual with polyps (III10) and the wild-type sequence for II7 (CRC at the age of 83 years) and the two controls of the family (III3 and IV3), respectively (Supplementary Figure S1a).

### 2.2. FCVPPv2 Analysis of Non-Coding Variants Prioritized a 5′UTR Variant in HDAC5 Gene

In agreement with the high proportion of non-coding DNA in the human genome, 674 of the 783 pedigree-filtered variants (86%) were located in the non-coding sequence such as intronic and intergenic regions, 1 kb downstream and upstream regions, the 5′UTR, 3′UTR and non-coding RNAs (ncRNAs), respectively (Figure 2). Screening of the 174 upstream/5′UTR as well as downstream/3′UTR variants, excluding intronic, intergenic and ncRNAs variants, for an updated PHRED-like CADD score ≥ 10 resulted in 38 variants. Filtering with conservational scores narrowed down this number to 8 5′UTR and 18 3′UTR variants. Application of non-coding scores derived from SNPnexus revealed that all 8 5′UTR variants reached at least 50% of the cut-off values, whereas 2 out of 18 3′UTR variants were excluded due to insufficient non-coding scores. The remaining 24 non-coding candidates were further evaluated for the presence of specific regulatory elements such as TFBS (transcription factor binding sites) and CpG islands for 5′UTR and miRNA binding sites for 3′UTR variants. Since all 5′UTR candidates showed either a CpG island or a TFBS identified by SNPnexus, CADD v1.4 or Bedtools intersect function, our analysis resulted in 8 top 5′UTR variants, summarized in Table 2.

Out of the remaining 16 3′UTR variants only 8 were annotated with a predicted miRNA target site by the Bedtools intersect function and with a mirSVR score ≤ −0.1, shortlisted as the top 3′UTR variants in Table 3.

Checking the shortlisted variants for their involvement in molecular mechanisms of colorectal carcinogenesis such as Wnt and Notch signaling pathways, which are generally known to play a crucial role in CRC initiation [29], revealed that the *HDAC5* gene was implicated in colorectal carcinogenesis by upregulating the Delta-like 4 ligand (DLL4), a vascular specific Notch ligand essential for tumor angiogenesis [30,31]. The potentially pathogenic role of HDAC5 in CRC was clinically confirmed by a further study showing the upregulation of HDAC5 protein in patients with early colon field carcinogenesis [32]. Therefore, the short-listed 5′UTR variant of the *HDAC5* gene (17_42200942_T_G) was considered as a promising cancer-predisposing candidate. As the variant was annotated to be located at an active transcription start site according to ChromHmm (*cHmmTssA, Score* = 0.984) and Segway (*TSS*) (Table 2), an activating impact of the 5′UTR variant on *HDAC5* gene expression was hypothesized. The location of the variant in a CpG island and multiple TFBSs as well as the high PHRED-like CADD score of 21.9 supported the potential functional role of the identified *HDAC5* variant in cancer predisposition, leading to its final prioritization (Figure 2). Pedigree segregation of the prioritized variant was checked by IGV and further confirmed by targeted Sanger sequencing showing the wild-type sequence for family members III3, III10 and IV3 and the heterozygous variant (T → G) for II7, III7 and III8, respectively (Supplementary Figure S1b).

### 2.3. Allele Frequency in a Large Familial CRC Cohort

Custom-made Taqman assays for screening of the *APCDD1* and *HDAC5* variants in 1705 familial CRC cases and 1674 healthy elderly individuals from Poland confirmed the variants in the family. Screening of *APCDD1* resulted in identifying the variant in an additional 8 familial CRC cases and 2 healthy individuals (odds ratio (OR) = 4.44, 95% confidence interval (CI) = [0.96; 20.56], *p* = 0.06). Additionally, one individual, who originally was in the healthy control group but developed CRC at the age of 55 years, was heterozygous for the variant. That increased the OR to 4.93 (95%CI = [1.08; 22.53], *p* = 0.04). The existence of the heterozygous *APCDD1* variant was confirmed by Sanger sequencing in all positive samples. All CRC patients were diagnosed at ages between 30 and 64 years and had at least one family member diagnosed with CRC, in some cases also with other cancers such as breast, cervical, female genital tract, kidney and lung cancer and leukemia. The sampling ages of the two healthy individuals were 55 years and 80 years and they had no family history of any cancer. No other familial CRC cases showing the *HDAC5* variant were identified.

### 2.4. APCDD1 Variant Did Not Show a Significant Effect on Proliferation of HEK293T and HT-29 Cells

In order to investigate the functional impact of the identified *APCDD1* variant, CCK-8 proliferation assays were conducted for *pAPCDD1^WT^* and *pAPCDD1^MUT^* using HEK293T and HT-29 cell lines. We did not find any significant difference in viable cell numbers between *pAPCDD1^WT^* and *pAPCDD1^MUT^* transfected cells at any measured time point (*p* = 0.05, Supplementary Figure S2). These results indicate an improbable proliferative impact of the variant in HEK293T cells as well as in colon cancer cells HT-29, excluding the *APCDD1* variant as a sole potentially cancer-predisposing candidate in the studied family.

### 2.5. 5′ UTR Variant of HDAC5 Gene Enhances Promoter Activity

**Figure 3:**
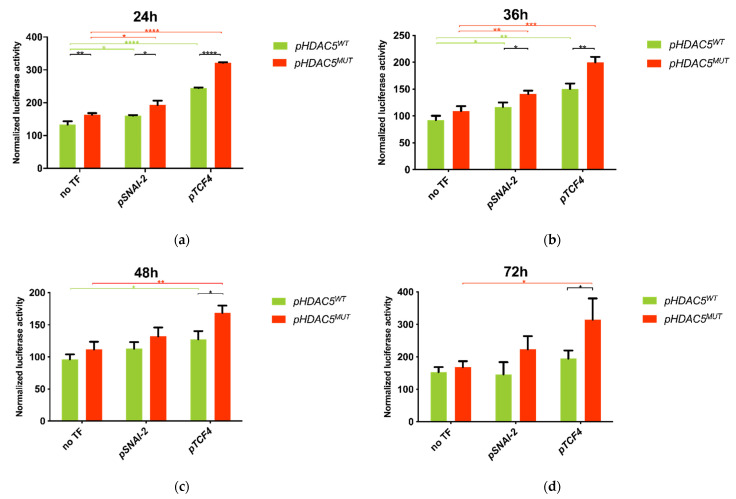
*pHDAC5^MUT^* shows a significantly increased luciferase activity compared to *pHDAC5^WT^.* Dual luciferase reporter assays performed for pGL4.10-*HDAC5^WT^* and pGL4.10-*HDAC5^MUT^* reporter constructs co-transfected with *pSNAI-2* or *pTCF4* or no transcription factor (TF) into HEK293T cells. Luciferase activity was measured at four different time points (**a**–**d**) and normalized to the empty pGL4.10 reporter vector. Each bar represents the mean of three independent experiments with standard deviation. *pHDAC5^MUT^* shows a significantly increased luciferase activity compared to *pHDAC5^WT^*. Co-transfection of *pTCF4* further enhanced the promoter activity of *pHDAC5^MUT^* significantly. *—*p* < 0.05, **—*p* < 0.01, ***—*p* < 0.001, ****—*p* < 0.0001.

To test our hypothesis that the identified 5′UTR variant contributes to increased *HDAC5* expression at transcriptional level, we performed luciferase reporter assays in HEK293T cells with both *pHDAC5*^WT^ and *pHDAC5^MUT^*. The results of the reporter assays revealed a consistently higher luciferase activity (*R* = *L_F_/L_R_*) of cells transfected with *pHDAC5*^MUT^ compared to *pHDAC5*^WT^ after normalization to pGL4.10 vector (*R_E_* = 1), respectively (Figure 3). Despite the overall increasing tendency, only the first time point after 24 h post transfection resulted in a significant fold change in activity (Δ*FA_MUT/WT_* = 122.64%, *t*-test *p* < 0.01), whereas the later time points showed no significant difference between *pHDAC5^WT^* and *pHDAC5^MUT^* using *t*-tests at a significance level of *α =* 0.05. Since both plasmids, *pHDAC5^WT^* and *pHDAC5^MUT^*, only differ in the variant of interest (*HDAC5*: 17_42200942_T_G), the detected increase of luminescent signal can be traced back to the variant itself causing the enhanced promoter activity.

### 2.6. 5′ UTR Variant of HDAC5 Disrupts SNAI-2 and TCF4 Transcription Factor Binding Sites

Analysis of TFBS predicted by Jaspar2020 with the default relative profile score threshold of 80% resulted in the identification of 22 newly created TFBS (only present in *HDAC^MUT^*) and 43 TFBS destroyed by the variant (only present in *HDAC5^WT^*). The overall 65 identified TFBS differing between *HDAC5^WT^* and *HDAC^MUT^* were found to be targeted by 51 different transcription factors. Further restriction of the relative score to 85%, which is referring to the likelihood sequence for the motif, narrowed down the number of identified transcription factors to 17, targeting 21 different TFBS (Table 4). Literature mining showed an association of two specific transcription factors with colorectal carcinogenesis: SNAI-2 and TCF4 have been shown to be involved in Wnt as well as in Notch pathway and were thus considered as promising candidates for upregulation of *HDAC5* promoter activity in further luciferase reporter assays [33,34]. According to Jaspar 2020, TCF4 was annotated to bind at 25 TFBS within the cloned *HDAC5* sequence of which 2 were disrupted in *HDAC^MUT^*, whereas SNAI-2 was reported to bind at 13 TFBS with 1 binding site disrupted in *HDAC^MUT^*.

### 2.7. Co-Transfection of HDAC5 and TCF4 Increases Promoter Activity Due to 5′UTR Variant of HDAC5 Gene

To investigate the effect of potential regulatory transcription factors (SNAI-2 and TCF4) on the promoter activity of *HDAC5* gene, HEK293T cells that do not express these transcription factors endogenously were co-transfected with the respective expression vectors of *pSNAI-2* or *pTCF4* followed by luciferase reporter assays. The results showed an enhanced promoter activity for almost all measured time points after expression of the transcription factors compared to the cells only transfected with respective *pHDAC5^WT^/pHDAC5^MUT^* vectors, respectively (Figure 3).

Co-transfection of *pSNAI-2* led to a significant increase in luciferase activity of both *pHDAC5^WT^* and *pHDAC5^MUT^* cells compared to respective cells not expressing *pSNAI-2,* showing similar fold changes at 24 h and 36 h (*t*-test *p* < 0.05). To see if the described effect is partly mediated by the identified variant, we next compared the luciferase activity between *pHDAC5^WT^* and *pHDAC5^MUT^* cells co-transfected with *pSNAI-2*. This resulted in a significant fold change in promoter activity after 24 h and 36 h of transfection (Δ*FA_MUT,S/WT,S_ =* 120.44% (24 h), 120.55% (36 h), *t*-test *p* < 0.05). Nevertheless, the fold change between *pHDAC5*^WT^ and *pHDAC5^MUT^* cells not expressing *pSNAI-2* was observed to be similar (Δ*FA_MUT/WT_* = 122.64% (24 h), *t*-test *p* < 0.01), which was also mirrored by the overlapping 95% confidence intervals of differences between *pHDAC5^WT^* and *pHDAC5^MUT^* cells respectively with or without *pSNAI-2* expression (95% *CI_MUT,S-WT,S_* = [10.90; 54.57]; 95% *CI_MUT-WT_* = [12.37; 47.96]). In summary, SNAI-2 transcription factor increased *HDAC5* promoter activity independent of the variant of interest after 24 h and 36 h of transfection.

Co-transfection of *pTCF4* expression vector also led to enhanced luciferase activity in both *pHDAC5^WT^* and *pHDAC5^MUT^* cells compared to respective cells not expressing *pTCF4*, whereas a higher effect on the cells carrying the mutated sequence was observed. This stronger enhancement of *pHDAC5^MUT^* promoter activity by *pTCF4* expression is well reflected in the comparison between *pHDAC5^WT^* and *pHDAC5^MUT^* cells: after co-transfection of *pTCF4, pHDAC5^MUT^* cells showed a significantly higher promoter activity compared to *pHDAC5^WT^* cells at all four measured time points, reaching its maximum at 72 h (Δ*FA_MUT,T/WT,T_ =* 131.66%, 132.99%, 132.05%, 161.52%, respectively, *t*-test *p* < 0.05). In contrast to that, fold changes in promoter activity of *pHDAC5^MUT^* compared to *pHDAC5^WT^* without any co-transfection never exceeded the maximum value of 122.64% (24 h post transfection) and were thus lower than in *pTCF4* co-transfected cells. Consistently, the difference between means of *pHDAC5^WT^* and *pHDAC5^MUT^* promoter activity was detected to be at least 2.5-fold higher in cells co-transfected with *pTCF4* compared to cells not expressing *pTCF4*; here again the maximum of differences between means was reached after 72 h, showing an almost 8-fold value (Δ*R_MUT,T-WT,T_ =* 119.9; Δ*R_MUT-WT_* = 15.29). Furthermore, comparison of the 95% confidence intervals of differences between *pHDAC5^WT^* and *pHDAC5^MUT^* revealed no overlapping for cells with or without *pTCF4* expression 24 h post transfection (95% *CI _MUT,T-WT,T_ =* [73.44; 81.18]; 95% *CI_MUT-WT_* = [12.37; 47.96]). Summing up the described results, the enhancing effect of *pTCF4* on *HDAC5* promoter activity can be partly traced back to a mechanism depending on the prioritized 5′UTR variant.

The dependency of TCF4-mediated promoter activity enhancement on the identified *HDAC5* variant was confirmed in a second experiment of luciferase reporter assays, focusing only on the comparison of *pTCF4* co-transfected cells at the same 4 time points: Fold changes in promoter activity of *pHDAC5^MUT^* compared to *pHDAC5^WT^* even reached values between 368.82% and 405.05% at all measured time points. Two-tailed *t*-tests resulted in extreme significance of promoter activity increase between *pHDAC5^WT^* and *pHDAC5^MUT^* cells (*p* < 0.0001).

## 3. Discussion

By applying our in-house developed FCVPPv2 on a CRC-affected family, we were able to prioritize two novel heterozygous germline variants, a coding variant in *APCDD1* and a non-coding variant in the *HDAC5* gene. The *APCDD1* variant was identified in 8 additional familial CRC cases, 1 CRC case without family history and in 2 healthy elderly individuals without cancer family history, leading to a 4.9-fold increased CRC risk for the variant carriers (*p* = 0.04), while no other *HDAC5* variants were identified among the 1705 familial CRC cases. Cell proliferation assays indicated an insignificant proliferative impact for the *APCDD1* variant and luciferase reporter assay results showed an increased promoter activity by the 5′UTR variant of the *HDAC5* gene.

APCDD1 was first identified as a direct Wnt target of the *β*-catenin/TCF4 transcription complex by Takahashi et al. who further reported increased APCDD1 expression in primary colon cancer tissue compared with corresponding healthy tissue [25]. In contrast, a study by De Sousa et al. showed that increased expression was restricted only to adenoma stages and not observed in carcinoma stages, and that Wnt target genes such as *APCDD1* were epigenetically silenced by promoter methylation in different colon cancer cell lines. Since re-expression of APCDD1 was further associated with decreased Wnt signaling levels, the authors explained these observations in CRC by the known negative feedback regulation of Wnt signaling driven by several target genes including *APCDD1* [35,36]. Showing decreased levels of *β*-catenin and Wnt target genes upon APCDD1 expression, Ordóñez-Morán et al. confirmed its role as a Wnt inhibitor and further proposed APCDD1 as a potential tumor suppressor in CRC [37]. Accordingly, both mentioned studies found a correlation of high APCDD1 expression with favorable prognosis for CRC patients [35,37]. Though a cell growth-promoting function was reported by Takahashi et al. for APCDD1 in vitro and in vivo using colon cancer LoVo cells [25], cell proliferation assays of this study using HEK293T and colon cancer cells HT-29 could not confirm the postulated proliferative impact of wild-type APCDD1 nor of the identified *APCDD1* variant. Based on the potential tumor suppressor role of APCDD1 and the variant being relatively common among Polish CRC cases, the identified APCDD1 variant could be considered to play a possible role in colorectal carcinogenesis, though our cell proliferation experiments did not show any difference between the wild-type and mutated HT-29 cells.

On the other hand, our study showed that implementation of the identified 5′UTR variant of the *HDAC5* gene increased the promoter activity. Experimental confirmation of pedigree segregation as well as the established functional role of HDAC5 in colorectal carcinogenesis supported a role for the 5′UTR variant in CRC predisposition: HDAC5 plays a crucial part in epigenetic modulation of gene expression. By removing acetyl groups from the *N*-acetyllysine residues of histones, HDACs are able to enhance chromatin condensation, leading to transcriptional repression of genes [38]. Thus, dysregulation of HDACs induces chromatin rearrangement possibly affecting tumor suppressor genes or oncogenes which may explain the well-established association of HDACs with carcinogenesis of various malignancies such as CRC, medulloblastoma [39] or hepatocellular carcinoma [40]. In particular, upregulation of Notch ligand DLL4 and the possibly resulting activation of Notch pathway and angiogenesis could represent an important carcinogenic mechanism induced by HDAC5 in CRC. Furthermore, HDAC inhibitors such as azaindolylsulfonamides have already been investigated in CRC xenografts and have shown promising results in tumor growth suppression [41].

We showed an enhancing impact of the identified non-coding variant on *HDAC5* promoter activity with the help of luciferase reporter assays. A further upregulation of promoter activity by TCF4 expression, especially in cells carrying the mutation, could implicate TCF4 as a potential regulator of *HDAC5* expression partly depending on the inserted variant. Since implementation of the *HDAC5* 5′UTR variant leads to loss of TCF4 binding sites, the described enhancement could be traced back to the reduction of a potentially repressive function of TCF4 on *HDAC5* promoter activity at these specific sites. The enhancing effect of TCF4 as well on promoter activity of cells not carrying the mutation might be explained by further, still unexplored regulatory mechanisms as additional molecular interactions of TCF4 transcription factor with *HDAC5* promoter sequence. This may be supported by the identification of 23 additional TFBS targeted by TCF4 within the cloned *HDAC5* sequence. The results of this work indicate the involvement of TCF4 transcription factor in regulation of *HDAC5* gene expression, resulting in HDAC5 upregulation and potentially promoting colorectal carcinogenesis in this family. 

The proposed regulating impact of TCF4 on *HDAC5* gene expression in the studied family with CRC aggregation is further supported by the generally established role of TCF4 in colorectal carcinogenesis. As part of the Wnt signaling pathway, TCF4 was shown to form the β-catenin/TCF4 transcription complex in the nucleus and induce gene expression of Wnt targets such as *MYC* [33,42,43,44], a known oncogene overexpressed in CRC [45,46,47]. As reported by Hatzis et al., genes upregulated by TCF4 are involved in cell proliferation, transcription, cell adhesion, negative regulation of programmed cell death, establishment and maintenance of chromatin [48]. Furthermore, TCF4 has been reported as a negative prognostic factor in CRC and is associated with shorter overall survival [49]. Although the molecular mechanisms leading to further *HDAC5* upregulation after the assumed TCF4 binding site loss are not yet fully understood, the described carcinogenic role of TCF4 in CRC is supported by our results and supports reciprocally the postulated CRC promoting function of *HDAC5* gene. A possible explanation approach for the underlying molecular mechanisms may consider the known dual regulatory role of TCF4 depending on the interaction with either transcriptional co-repressor (such as Groucho/transducin-like enhancer of split (Gro/TLE) family members and HDACs) or co-activator complexes (such as β-catenin and SMADs) [50,51,52,53,54,55,56].

The results of our functional experiments provided further evidence for the application of the FCVPPv2 to families with cancer aggregation and confirmed our pipeline’s significance in the prioritization of both the coding and non-coding variants. The integration of a variety of annotation tools by the FCVPPv2 enables the identification of functionally important coding regions and regulatory elements in the non-coding sequence of genes. Regarding the prediction of TFBSs modified by the prioritized variant, CADD v1.4 identified a relatively high number of overlapping ChIP-seq TFBSs whereas SNPnexus and the intersect function of Bedtools did not reveal any. The differing results for predicted TFBSs could be traced back to the different databases each tool is based on and different cell lines used in the studies. Thus, the synergetic application of all tools within the FCVPPv2 with subsequent integration of their respective predictions could be considered as a good approach for an all-encompassing analysis of TFBSs.

## 4. Materials and Methods

### 4.1. Patient Samples

A family with 8 confirmed CRC cases in 3 generations was identified at the Department of Genetics and Pathology, Pomeranian Medical University in Szczecin, Poland. Blood samples were collected from 3 CRC cases, 2 siblings who were diagnosed with CRC at the age of 52 and 35 years and their aunt who developed CRC at the age of 83, 1 individual with polyps diagnosed at the age of 56, 59 and 71 years and 2 unaffected family members (Figure 1). The study was approved by the Bioethics Committee of the Pomeranian Medical Academy in Szczecin (No: BN-001/174/05). All participating individuals gave informed consent.

### 4.2. Whole Exome Sequencing and Variant Evaluation

Genomic DNA was isolated using a modified Lahiri and Schnabel method [57] and WES was performed for 3 CRC cases (II-7, III-7, III-8), a family member with polyps (III-10) and 2 unaffected family members (III-3, IV-3) using Illumina-based small read sequencing. Mapping to reference human genome (assembly version Hs37d5) was performed using BWA [58] and duplicates were removed using Picard (http://broadinstitute.github.io/picard/, accessed on 22 January 2021).

### 4.3. Variant Calling, Annotation and Filtering

Single nucleotide variants (SNVs) were detected by using SAM tools [59] and indels by using Platypus [60]. Variants were annotated using ANNOVAR [61], 1000 Genomes project [62], dbSNP [63] and Exome Aggregation Consortium (ExAC) [64]. Variants with a quality score of greater than 20 and a coverage of greater than 5×, SNVs that passed the strand bias filter (a minimum one read support from both forward and reverse strand) and indels that passed all the Platypus internal filters were retained. With respect to the 1000 Genomes project Phase 3, non-TCGA ExAC data [64], NHLBI-ESP6500 and local datasets variants with minor allele frequency (MAF) less than 0.1% in the European population were selected. A pairwise comparison of shared rare variants among the family was performed to check for sample swaps and family relatedness.

### 4.4. Variant Filtering According to FCVPPv2

The Familial Cancer Variant Prioritization Pipeline version 2 (FCVPPv2) was applied for evaluation of identified variants as described below and summarized in Figure 2 [14].

#### 4.4.1. Familial Segregation of the Cancer Predisposing Variants

Considering the probability of carrying the cancer-predisposing variant for each analyzed family member, they were classified as cases and controls according to the presence or absence of CRC. Generally, all affected family members should carry the variant of interest, with the following exception: Since the typical age of onset in hereditary CRC patients is considered to be lower than in the general population, such as 45 years in HNPCC families [65] compared to 63 years in sporadic CRC [66], family member II-7 developed CRC at a relatively high age of 83 years, atypical for familial inheritance. Therefore, she could be considered as a phenocopy in this family expressing the phenotypic disease, but not the underlying genotype. Whereas II-7 could possibly but not definitely carry the variant of interest, family members III-7 and III-8 are considered as certain cases and thus carriers of the variant due to their young age of onset typical for familial CRC. The unaffected family members III-3 and IV-3 should not show the cancer-predisposing variant of interest and are thus defined as controls in this family. On the other hand, family member III-10 was diagnosed with multiple colorectal polyps at the age of 56, 59 and 71 years. Since colorectal polyps could be a preliminary stage of CRC, especially when recurrent, III-10 could be considered as a possible carrier of the variant. Based on these definitions, the identified variants were filtered according to their presence in the cases, absence in the controls and presence or absence in the old-age CRC case II-7 and the polyp carrier.

#### 4.4.2. Analysis of Coding Variants

All variants were ranked using the Combined Annotation Dependent Depletion (CADD) tool v1.3 [67]; evolutionary conservation scores: Genomic Evolutionary Rate Profiling (GERP > 2.0), PhastCons (>0.3) and PhyloP (≥3.0) [68,69,70]; intolerance scores based on allele frequency data from our in-house datasets, from ESP [71] and ExAC supplemented by the ExAC-derived Z-score [72]; and deleteriousness scoring tools accessed from dbNSFP v3.0 (database for nonsynonymous SNVs’ functional predictions) [73]. The variants should reach a PHRED-like CADD-score of ≥ 10 and fulfill at least 2 out of 3 conservational scores, 3 out of 4 intolerance scores and at least 60% of all 12 deleteriousness scores to be taken into account for further analysis. The final exonic candidates were further screened by considering the allele frequency in the non-Finnish European population in the latest version of gnomAD database (https://gnomad.broadinstitute.org/, accessed on 22 January 2021), the potential impact of amino acid substitutions with the help of Snap2 (https://rostlab.org/services/snap2web/, accessed on 22 January 2021), the prediction of cancer drivers by Cancer Genome Interpreter (https://www.cancergenomeinterpreter.org/, accessed on 22 January 2021) and the recent literature for reported gene–cancer relations and potentially cancer-related protein functions [74,75]. 

#### 4.4.3. Analysis of Non-Coding Variants

Non-coding variants were analyzed with the updated version of CADD (CADD v1.4) that provides comprehensive information about the functional importance of non-coding regions by integrating a variety of scoring tools such as transcription factor binding sites (TFBS) located in the 5′UTR and 1 kb flanking region upstream of transcription start sites, mirSVR for ranking putative microRNA target sites [76], chromHmm and Segway that provide information about the biological function and active regulatory regions based on large-scale functional genomics datasets such as ChIP-seq data [26,28]. The variants were also analyzed by SNPnexus for identification of CpG islands and TFBS and for annotation of the functional impact of all non-coding variants [19]. Variants of the 5′UTR and 1 kb upstream region as well as 3′UTR and 1 kb downstream region were scanned for potential regulatory elements by means of Bedtools intersect function and respective databases: the FANTOM5 consortium and the super-enhancer archive (SEA) were used for identification of promoters, enhancers or super-enhancers [77] and Targetscan 7.0 was used for identification of microRNA target sites [78].

The literature was checked for any gene–cancer relations and potentially cancer-related protein functions of the top non-coding candidates.

### 4.5. Analysis of Transcription Factor Binding Sites

By uploading the wild-type sequence (*HDAC5^WT^*) and the sequence containing the variant in the 5′UTR of *HDAC5* gene (*HDAC5^MU^*^T^) to Jaspar2020, potential TFBS were predicted and compared [79].

### 4.6. Variant Validation with IGV

Sequencing data of all prioritized variants were checked for correctness using the Integrative Genomics Viewer (IGV), a visualization tool for interactive exploration of large, integrated genomic datasets [80]. By this means, the identified variants were validated and the confidence in variant calls was increased.

### 4.7. Confirmation of Familial Segregation by Sanger Sequencing

Polymerase chain reaction (PCR) was performed to amplify the 5′UTR of *HDAC5* gene (ENST00000225983.6) from DNA of the family members by using HotStarTaq DNA Polymerase (Qiagen, #203205) and following the manufacturer’s instructions. The primers were designed with Primer3 v.0.4.0 (http://bioinfo.ut.ee/primer3-0.4.0/, accessed on 22 January 2021) on 12 April 2019: HDAC5 forward 5′-gggggtctgggtctattttt-3′, reverse 5′-GAAGGGGCAAATCAGACAAC-3′. PCR was run at an annealing temperature of 62 °C with 5% dimethylsulfoxide (DMSO). The amplicons were validated by gel electrophoresis and purified with ExoSAP purification kit according to the manufacturer’s instructions. Sequencing reaction was performed with BigDye Terminator v3.1 Ready Reaction Cycle Sequencing kit (Thermo Fisher Scientific, Darmstadt, HE, Germany, #4337455). The electrophoretic profiles of *HDAC5* sequences were analyzed manually.

### 4.8. Screening of Large Case and Control Cohorts

In order to determine the allele frequency of the *HDAC5* and *APCDD1* variants, 1705 familial CRC cases and 1674 healthy elderly individuals without cancer family history were checked using custom-made Taqman assays. The existence of heterozygous variants was confirmed by Sanger sequencing.

### 4.9. PCR-Based Cloning of Gene Reporter Constructs

Cloning primers were designed using Primer3 v.0.4.0 (http://bioinfo.ut.ee/primer3-0.4.0/, accessed on 22 January 2021) on 12 April 2019 for the 5′UTR including the variant of interest as well as the first exon and part of the following intron of *HDAC5* gene (ENST00000225983.6). Adding specific restriction sites of Kpn I or Hind III and a 5′ leader sequence of 6 bp resulted in the following primer pair: forward 5′-TAAGCAGGTAC^C gcaccaaagtcagggaagtc-3′; reverse 5′-TGCTTA^AAGCTTgaaggggcaaatcagacaac-3′. PCR was performed with an annealing temperature of 59 °C with 5% DMSO to amplify the required *HDAC5* insert with a total length of 1116 bp from human DNA. Digestion of the PCR amplicon and the promoter-less pGL4.10[*luc2*] vector purchased from Promega (#E6651) was performed using FastDigest Kpn I (Thermo Fisher Scientific, FD0524), FastDigest Hind III (Thermo Fisher Scientific, #FD0504) and FastDigest Buffer 10x (Thermo Fisher Scientific, #B64) according to the manufacturer’s instructions. The digested products were validated by gel electrophoresis and extracted with Monarch^®^ DNA Gel Extraction Kit (New England BioLabs, Frankfurt, HE Germany, #T1020S). Ligation of the digested *HDAC5* gene insert (1099 bp) and pGL4.10 vector (4210 bp) was done by using Quick Ligation™ Kit (New England BioLabs, #M2200S). The ligated product pGL4.10-*HDAC5* (5309 bp), illustrated in Supplementary Figure S3, was again validated by gel electrophoresis and extracted with Monarch^®^ DNA Gel Extraction Kit (New England BioLabs, #T1020S) for further use as the wild-type pGL4.10-*HDAC5* (*pHDAC5^WT^*) construct. The mutant pGL4.10-*HDAC5* (*pHDAC5^MUT^*) construct was created by site-directed mutagenesis using QuikChange II XL Site-Directed Mutagenesis Kit (Agilent, Waldbronn, BW, Germany, #200521) according to the manufacturer’s instructions and the following primer pair: forward 5′-gcggcagcaccgcctcgacggct-3′, reverse 5′-agccgtcgaggcggtgctgccgc-3′, designed based on Agilent QuikChange Primer Design (https://www.agilent.com/store/primerDesignProgram.jsp, accessed on 22 January 2021) on 28 November 2020. Both plasmids, *pHDAC5^WT^* and *pHDAC5^MUT^*, were confirmed by Sanger sequencing.

### 4.10. Cloning of SNAI-2, TCF4 and APCDD1

Human pENTR223-*SNAI-2* (#172707094), pENTR223-*TCF4* (#107260711) and pENTR223-*APCDD1* clones (GPCF, #115154469) purchased from the Genomics and Proteomics Core Facility of the DKFZ Heidelberg (GPCF) were cloned into pDEST26 vector using Gateway™ LR Clonase™ II Enzyme mix (Thermo Fisher Scientific, #11791020). The identified variant was introduced into pDEST26-*APCDD1* vector (*pAPCDD1*) using QuikChange II XL SDM Kit and the following SDM primers designed with Agilent QuikChange Primer Design: forward 5′-gggtgagccagcactgtgaggtgcg-3′, reverse 5′-cgcacctcacagtgctggctcaccc-3′. All sequences were confirmed by Sanger sequencing.

### 4.11. Plasmid Amplification and Extraction

Stellar chemically competent cells (Takara, Saint-Germain-en-Laye, France, #636763) were used for transformation of *pHDAC5^WT^*, *pSNAI-2, pTCF4* and *pAPCDD1^WT^,* whereas *pHDAC5^MUT^* and *pAPCDD1^MUT^* were transformed into XL10-Gold Ultracompetent cells (Agilent, #200314) after site-directed mutagenesis. Plasmid extraction was performed using PureLink™ HiPure Plasmid Midiprep Kit (Thermo Fisher Scientific, #K210004).

### 4.12. Cell Line and Culture Conditions

Human embryonic kidney 293 (HEK293T) cells and human colon cancer cells HT-29 were a kind gift from Peter Krammer’s lab (DKFZ) and cultured in RPMI. Using Harmonizome, a database of processed datasets about genes and proteins, endogenous expression levels of proteins for HDAC5, SNAI-2 and TCF4 were checked and ruled out in HEK293T cells [81].

### 4.13. Cell Proliferation Assay—APCDD1

HEK293T and HT-29 cells were seeded in 24-well plates and 24 h later transfected with either 150 ng of *pAPCDD1^WT^*, *pAPCDD1^MUT^* or pDEST26 vector as negative control. Merck’s Cell Counting Kit-8 (CCK-8, Darmstadt, HE, Germany #96992) was used for quantitation of viable cell numbers in proliferation and cytotoxicity assays at four different time points: 0 h, 24 h, 48 h and 72 h post transfection. Briefly, a 100 μL cell suspension of each well was treated with 10 μL CCK-8 solution and incubated for 1 h at 37 °C. The absorbance was measured at 450 nm using a microplate reader and the number of viable cells was calculated based on a standard curve. By comparing the numbers of viable cells at different time points and the respective growth curves between *pAPCDD1^WT^* and *pAPCDD1^MUT^*, the proliferative impact of the implemented *APCDD1* variant (p.R299H) could be estimated for each cell line.

### 4.14. Luciferase Reporter Assay—HDAC5

HEK293T cells were seeded in 48-well plates and 24 h later transfected with 100 ng of *pHDAC5*^WT^ or *pHDAC5^MUT^* as a test reporter, 10 ng renilla as the control reporter and 25 μL Lipofectamine 2000 (Thermo Fisher Scientific, #11668030). Negative controls were considered by including cells transfected with promoter-less pGL4.10 vector (EMPTY, E). For investigating the impact of SNAI-2 and TCF4 transcription factors, cells were co-transfected with 20 ng of *pSNAI-2* or *pTCF4* expression vector and the corresponding negative controls were included: pGL4.10 vector in combination with each expression vector (*pSNAI-2* or *pTCF4*) and *pHDAC5^MUT^* with the empty expression vector pDEST26. Luciferase assays were conducted using the dual-luciferase reporter assay system (Promega, Walldorf, BW, Germany, #E1910) at four different time points: 24 h, 36 h, 48 h and 72 h post transfection. Since renilla luminescence is measured for vector normalization, the relative ratio *R* of firefly luminescence *L_F_* to renilla luminescence *L_R_* (*R* = *L_F_*/*L_R_*) was calculated and later referred to as luciferase activity. After normalizing *R* values to the empty promoter-less pGL4.10 vector (*R_E_ = 1*), the ratios were compared between *pHDAC5^WT^* and *pHDAC5^MUT^* for each condition. For this purpose, we calculated fold changes in promoter activity (Δ*Fold Activity _MUT/WT_* = Δ*FA_MUT/WT_* = *R_MUT_/R_WT_*) as well as two-tailed *t*-tests at a significance level of *α* = 0.05. All experiments were conducted in triplicates and repeated at least thrice.

## 5. Conclusions

Application of the FCVPPv2 on a CRC-affected family identified a novel missense variant in the *APCDD1* gene and a 5′UTR variant in the *HDAC5* gene as potentially cancer predisposing. While the *APCDD1* variant was relatively common among Polish CRC cases (*AF* = 0.003) and increased the risk of CRC 4.9-fold for the variant carriers, it did not seem to affect cell proliferation in vitro. On the other hand, the *HDAC5* variant shows a low allele frequency in all world populations as well as an enhancing effect on *HDAC5* promoter activity in luciferase reporter assays and thereby on *HDAC5* gene expression. Our findings support the importance of taking into account both coding and non-coding variants in cancer predisposition, population screening and functional validation of variants.

## Figures and Tables

**Table 1:** Overview of the top exonic missense variants prioritized in the studied CRC family. Chromosomal positions, pedigree segregation, allele frequencies in the non-Finnish European population (*NFE*), accessed on 28 November 2020, PHRED-like CADD (Combined Annotation Dependent Depletion v1.3) scores, conservational scores and the percentage of reached intolerance and deleteriousness scores are summarized for each variant. Snap^2^ results for the predicted amino acid changes with calculated effect scores and accuracies given in % as well as CGI predictions were accessed on 28 November 2020 and included in the table. Respective protein functions of the encoded gene products are derived from Genecards on 26 November 2020 [20]. *ND*—no driver mutation; .—not annotated.

Gene Name	Variant	Pedigree Segregation	Allele Frequency in NFE		CADD SCORE	Conservational Scores			Intolerance Scores (%)	Deleteriousness Scores * (%)	Amino Acid Change	Snap^2^		CGI	Protein Function
			ExAC	gnomAD		GERP	PhyloP	PhastCons				Score	Accuracy (%)		
APCDD1	18_10485580_G_A	III7, III8, III10	7.37 × 10^−5^	1.4 × 10^−4^	27.5	4.94	9.26	1	100	66.67	R299H	54	75	ND	Inhibition of Wnt signaling, controversial function
FLNC	7_128480709_G_A	III7, III8, III10	5.25 × 10^−4^	2.57 × 10^−4^	29.3	5.02	9.82	1	100	100	G553S	33	66	ND	Anchoring of membrane proteins for actin cytoskeleton
KCNH6	17_61613135_T_G	III7, III8, III10	4.51 × 10^−5^	6.16 × 10^−5^	23.4	3.39	3.96	1	100	91.67	L403V	6	53	ND	Regulation of neurotransmitter release, neuronal excitability, epithelial electrolyte transport
LSR	19_35741380_C_A	II7, III7, III8, III10	.	.	27.4	4.88	7.21	1	75	75	A139D	80	91	ND	Lipoprotein metabolism
MTX1	1_155181922_A_G	III7, III8, III10	9.25 × 10^−5^	1.52 × 10^−4^	23.3	3.64	2.89	1	66.67	66.67	Y228C	32	66	ND	Mitochondrial protein import
SDS	12_113836573_G_A	III7, III8, III10	4.59 × 10^−5^	5.44 × 10^−5^	23.4	4	5.69	1	75	100	T91I	46	71	ND	Serine and glycine metabolism, gluconeogenesis
ZW10	11_113607367_C_G	III7, III8, III10	5.99 × 10^−4^	6.43 × 10^−4^	34	6.17	7.49	1	75	75	A732P	70	85	ND	Chromosome segregation, mitotic checkpoint

* Following predictions given by deleteriousness scores were considered as favorable in our analysis: SIFT—Damaging (D); Polyphen2_HumDiv, Polyphen2_HumVar—Probably damaging (D) and Possibly damaging (P); LRT—Deleterious (D); MutationTaster—Disease causing (D) and disease causing automatic (A); MutationAssesor—High (H) and medium (M); FATHMM—Damaging (D); MetaSVM—Damaging (D); MetaLR—Damaging (D); Reliability Index ≥ 5; VEST3 ≥ 0.5; PROVEAN—Damaging (D).

**Table 2:** 5′UTR germline variants prioritized in the CRC family. Chromosomal position, pedigree segregation and allele frequencies in the non-Finnish European population (*NFE*), accessed on 28 March 2019, are listed for each variant. Promoters, enhancers, super-enhancers identified with Bedtools intersect function and SEA, FANTOM5 databases on 6 April 2019 are included. Overall deleteriousness, genomic conservation, chromatin state and location within transcription factor binding sites (TFBS) are annotated by CADD v1.4 on 30 March 2019. Further information on TFBS, CpG islands and the general functional impact in form of a summarizing percentage of positive non-coding scores are derived from SNPnexus on 31 March 2019. .—not annotated.

Gene Name	Variant	Pedigree Segregation	Allele Frequency in NFE		Bedtools Intersect ^I^	CADD v1.4									SNPnexus			
			ExAC	gnomAD NFE		CADD SCORE	Conservational Scores			Chromatin State			TFBS	TFBS Peaks ^IV^	Non Coding Scores ^V^ (%)	CpG Island	CpG Ratio ^VI^	TFBS
							PhastCons	PhyloP	GERP	ChromHMM ^II^ State	ChromHMM ^II^ Score	Segway ^III^						
CA4	17_58227298_G_C	III7, III8, III10	.	.	P:58227287 ..58227313,+	12.6	0.44	0.72	2.77	Enh Biv	0.24	TF0	1	4	50	61	0.89	.
CALM3	19_47105342_A_G	II7, III7, III8, III10	.	.	.	19.8	0.87	1.94	2.49	TssA	0.87	GS	1	1	50	.	.	.
HDAC5	17_42200942_T_G	II7, III7, III8	.	6.64 × 10^−5^	.	21.9	1.00	0.87	3.99	TssA	0.98	TSS	17	42	50	92	0.97	
PLAA	9_26947165_G_A	III7, III8, III10	.	6.49 × 10^−5^	P:26947129 ..26947212,-	22	0.99	0.92	4.8	TssA	0.94	TSS	41	61	83.3	72	0.86	NRF2
PPTC7	12_111021082_G_C	III7, III8, III10	.	2.69 × 10^−4^	SE: hg19_A549_2 12_111015565	16.5	1.00	3.85	4.29	TssA	0.98	TSS	26	50	50	83	1.13	.
TMEM 115	3_50396814_C_G	III7, III8, III10	.	1.69 × 10^−3^	.	18.1	1.00	1.89	4.82	TssA	0.82	GS	18	27	66.7	64	0.67	.
TPM2	9_35690678_C_T	III7, III8, III10	.	1.36 × 10^−3^	.	17.2	1.00	0.61	3.76	TssA	0.95	GE2	6	6	50	109	0.76	.
UBE2K	4_39699921_G_C	III7, III8, III10	.	1.16 × 10^−4^	.	17.0	1.00	1.39	4.47	TssA	0.93	TSS	14	25	66.7	89	1.01	.

^I^ Bedtools intersect: Promoters (P) are listed with their specific genomic position (P: start..end, strand). Super-enhancers (SE) are shown including information about the used reference genome, cell line and genomic position. ^II^ ChromHMM: The ChromHmm score shows the proportion of 127 cell types of the Roadmap Epigenomics project in a particular chromatin state with scores closer to 1 indicating more cell types in the particular chromatin state. The 15 chromatin states are defined as follows: TssA—Active transcription start site (TSS), TssAFInk—Flanking active TSS, TxFlnk—Transcribed at gene 5’ and 3’, Tx—Strong transcription, TxWk—Weak transcription, EnhG—Genic enhancers, Enh—Enhancers, ZNF/Rpts—ZNF genes and repeats, Het—Heterochromatin, TssBiv—Bivalent/Poised TSS/Enhancers, BivFlnk—Flanking bivalent TSS/Enhancer, EnhBiv—Bivalent enhancers, ReprPC—Repressed PolyComb, ReprPCWk—Weak Repressed PolyComb, Quies—Quiescent/low [26,27]. ^III^ Segway: Segway uses a genomic segmentation method to annotate the chromatin state based on multiple datasets of ChIP-seq experiments. The chromatin states can be annotated as follows: D—dead, F0/1—FAIRE, R0/1/2/4/5—Repressed Region, H3K9me1—histone 3 lysine 9 monomethylation, L0/1—Low zone, GE0/1/2—Gene body (end),TF0/1/2—Transcription factor activity, C0—CTCF, GS—Gene body (start), E/GM—Enhancer/gene middle, GM0/1—Gene body (middle), TSS—Transcription start site, ZnfRpts—zinc finger repeats [28]. ^IV^ TFBS peaks: The number of overlapping ChIP TFBS peaks summed over different cell types/tissue. ^V^ Non-coding scores: Following cut-offs were used for the interpretation of the non-coding scores derived from SNPnexus: FitCons Score ≥ 0.2 with a *p*-value ≤ 0.05; EIGEN > 0 (at least 1 of 2 scores); FatHMM > 0.5; GWAVA > 0.4 (at least 2 of 3 scores); DeepSEA > 0.5 (at least 2 of 3 scores); FunSeq2 > 3, ReMM > 0.5. ^VI^ CpG Ratio: The ratio of observed to expected CpG in island.

**Table 3:** 3′UTR germline variants prioritized in the CRC family. Chromosomal position, pedigree segregation and allele frequencies in the non-Finnish European population (*NFE*), accessed on 28 March 2019, are listed for each variant. miRNA target sites identified with Bedtools intersect function and Targetscan 7.0 databases on 6 April 2019 are included. Overall deleteriousness, genomic conservation, chromatin state and the likelihood of target mRNA downregulation predicted by mirSVR score are annotated by CADD v1.4 on 30 March 2019. The general functional impact of each 3′UTR variant is summarized by the percentage of positive non-coding scores derived from SNPnexus on 31 March 2019. .—not annotated.

Gene Name	Variant	Pedigree Segregation	Allele Frequency in NFE		Bedtools intersect ^I^			CADD v1.4								SNPnexus Non-Coding Scores ^IV^ (%)
			ExAC	gnomAD	miRNA Target Sites	Context Score ++ Percentile	Site Type	PHRED	Conservational Scores			Chromatin State			mirSVR-Score	
									verPhCons	verPhyloP	GerpN	ChromHMM ^II^ State	ChromHMM ^II^ Score	Segway ^III^		
ACTN2	1_236927298_T_A	III7, III8, III10	.	6.48 × 10^−5^	miR-450b-5p miR-891b	36 71	7mer-m8 7mer-1a	13.84	0.84	0.60	5.70	Quies	0.488	R2	−0.90	66.7
CCNT2	2_135714351_C_T	III7, III8, III10	.	.	miR-4670-3p miR-3606-5p	93 83	7mer-1a 8mer	15.46	1.00	3.28	5.65	Tx Wk	0.551	GE0	−1.11	83.3
CLK1	2_201717953_C_T	III7, III8	.	2.01 × 10^−3^	miR-4718 let-7c-3p	98 96	7mer-m8 7mer-m8	15.57	0.68	0.38	5.34	Tx	0.921	GE0	−1.21	85.7
DDX17	22_38881603_ACT_A	II7, III7, III8	.	2.84 × 10^−3^	miR-550b-3p	91	7mer-m8	14.91	0.66	0.97	4.36	Tx	0.858	F1	−0.35	100
FH	1_241661076_T_C	III7, III8, III10	.	3.89 × 10^−3^	miR-4294	94	7mer-m8	10.4	0.47	0.11	4.56	Tx	0.709	GE0	−0.96	71.4
LRRC8C	1_90180607_T_A	III7, III8, III10	.	2.59 × 10^−4^	miR-499a-5p miR-208-3p miR-4432 miR-8087	96 91 81 90	8mer 7mer-1a 7mer-1a 7mer-1a	21.8	1.00	3.21	6.17	Tx Wk	0.583	F1	−0.76	83.3
SEMA4B	15_90772811_G_A	III7, III8, III10	.	.	miR-3918 miR-3127-5p miR-506-5p miR-10a-3p	99 99 92 94	8mer 8mer 7mer-m8 7mer-1a	13.65	0.53	3.01	4.95	Tx	0.606	GE1	−1.27	83.3
TJP1	15_29993152_G_A	III7, III8	.	6.49 × 10^−5^	miR-654-3p	89	8mer	15.03	1.00	1.48	5.79	Tx	0.441	GE1	−0.80	83.3

^I^ Bedtools intersect: Using Bedtools intersect function and Targetscan 7.0 database, miRNA target sites were predicted for each variant. Multiple predicted miRNA target sites of one variant are listed separated with commas. The Context Score ++ Percentile shows the percentage of sites for the miRNA with a less favorable Context Score ++ and thus less repression capacity. Hence, a higher Context Score ++ Percentile indicates greater repression at a specific site by a miRNA compared to all sites of this miRNA family. The annotation of the site type gives information about the site efficacy in the seed region and thus about the different numbers of targets identified per miRNA. The order from the strictest to the least strict is as follows: 8mer > 7mer-m8 > 7mer-A1 > 6mer. ^II^ ChromHMM: ChromHmm shows the proportion of 127 cell types of the Roadmap Epigenomics project in a particular chromatin state with scores closer to 1 indicating more cell types in the particular chromatin state. The 15 chromatin states are defined as follows: TssA—Active transcription start site (TSS), TssAFInk—Flanking active TSS, TxFlnk—Transcribed at gene 5’ and 3’, Tx—Strong transcription, TxWk—Weak transcription, EnhG—Genic enhancers, Enh—Enhancers, ZNF/Rpts—ZNF genes and repeats, Het—Heterochromatin, TssBiv—Bivalent/Poised TSS/Enhancers, BivFlnk—Flanking bivalent TSS/Enhancer, EnhBiv—Bivalent enhancers, ReprPC—Repressed PolyComb, ReprPCWk—Weak Repressed PolyComb, Quies—Quiescent/low [26,27]. ^III^ Segway: Segway uses a genomic segmentation method to annotate the chromatin state based on multiple datasets of ChIP-seq experiments. The chromatin states can be annotated as follows: D—dead, F0/1—FAIRE, R0/1/2/4/5—Repressed Region, H3K9me1—histone 3 lysine 9 monomethylation, L0/1—Low zone, GE0/1/2—Gene body (end),TF0/1/2—Transcription factor activity, C0—CTCF, GS—Gene body (start), E/GM—Enhancer/gene middle, GM0/1—Gene body (middle), TSS—Transcription start site, ZnfRpts—zinc finger repeats [28]. ^IV^ Non-coding scores: Following cut-offs were used for the interpretation of the non-coding scores derived from SNPnexus: FitCons Score ≥ 0.2 with a *p*-value ≤ 0.05; EIGEN > 0 (at least 1 of 2 scores); FatHMM > 0.5; GWAVA > 0.4 (at least 2 of 3 scores); DeepSEA > 0.5 (at least 2 of 3 scores); FunSeq2 > 3, ReMM > 0.5.

**Table 4:** Summary of transcription factor binding sites (TFBS) identified with Jaspar2020 and relative profile score threshold of 85% on 30 November 2020. Matrix ID, names of targeting transcription factors, relative scores, start and end position, strand information and respective binding sequences are included. A relative score of 1 represents the maximum likelihood sequence for the motif. TFBS newly created by the variant and thus exclusively present in *HDAC5^MUT^* sequence are annotated as *MUT*, whereas TFBS destroyed by the variant and thus exclusively present in *HDAC5^WT^* sequence are annotated as *WT.*

Matrix ID	Name	Relative Score	Exclusive for *pHDAC5*	Start	End	Strand	Predicted Sequence
MA1631.1	ASCL1(var.2)	0.859965928	WT	710	722	-	cagcacctcctcg
MA0598.1	EHF	0.869445857	WT	711	718	-	acctcctc
MA0056.1	MZF1	0.854364496	WT	710	715	+	cgagga
MA0673.1	NKX2-8	0.857521064	WT	708	716	-	ctcctcgac
MA1558.1	SNAI-1	0.875024573	WT	712	721	+	aggaggtgct
MA0745.1	SNAI-2	0.891814443	WT	712	720	+	aggaggtgc
MA1563.1	SOX18	0.858931866	MUT	714	721	-	agcaccGc
MA0079.3	SP1	0.871792316	MUT	710	720	-	gcaccGcctcg
MA0079.3	SP1	0.862428729	WT	710	720	-	gcacctcctcg
MA0080.2	SPI1	0.865925519	WT	712	718	+	aggaggt
MA1566.1	TBX3	0.876483102	WT	714	723	+	gaggtgctgc
MA0806.1	TBX4	0.854062458	WT	715	722	+	aggtgctg
MA1567.1	TBX6	0.867675674	WT	714	723	+	gaggtgctgc
MA1648.1	TCF12(var.2)	0.868262172	WT	711	721	-	agcacctcctc
MA0522.3	TCF3	0.875470132	WT	711	721	-	agcacctcctc
MA0522.2	TCF3	0.869530427	WT	712	721	-	agcacctcct
MA0830.2	TCF4	0.851528821	WT	710	722	-	cagcacctcctcg
MA0830.1	TCF4	0.850775152	WT	712	721	-	agcacctcct
MA0003.1	TFAP2A	0.860276583	MUT	707	715	-	Gcctcgacg
MA0815.1	TFAP2C(var.3)	0.858234123	MUT	704	716	+	agccgtcgaggCg
MA0815.1	TFAP2C(var.3)	0.851532375	MUT	704	716	-	cGcctcgacggct

## Data Availability

Unfortunately, for reasons of ethics and patient confidentiality, we are not able to provide the sequencing data into a public database. The data underlying the results presented in the study are available from the corresponding author or from Asta Försti (Email: a.foersti@kitz-heidelberg.de).

## Supplementary Materials

The following are available online at https://www.mdpi.com/1422-0067/22/4/1837/s1, Figure S1: Representative electropherograms depicting the *APCDD1* and *HDAC5* variants identified in the studied CRC family, Figure S2: Cell proliferation assays conducted for *pAPCDD1^WT^* and *pAPCDD1^MUT^*, Figure S3: Graphical overview of pGL4.10-*HDAC5* reporter constructs, Table S1: Summary of family members analyzed in our study.
