# Supplementary material for: Whole Exome Sequencing Identifies APCDD1 and HDAC5 Genes as Potentially Cancer Predisposing in Familial Colorectal Cancer
Source: Int J Mol Sci. 2021 Feb 12;22(4):1837. doi: 10.3390/ijms22041837 (PMC7917948; doi:10.3390/ijms22041837)
Supplement: Supplementary file 1 [file ijms-22-01837-s001.pdf]

Table S1, Summary of family members analyzed in our study including the consideration of being a carrier of the cancer-causing variant. *CRC – colorectal cancer, P - colorectal polyps*

Classification	ID	Sex	Age at sampling	Age of onset of CRC	Age of onset of P	Considered as a carrier of the variant?
**Cases**	III7	male	-	52 x2		Yes
	III8	female	-	35	-	Yes
**Case / possible phenocopy**	II7	female	-	83	-	Yes/No
**Polyps**	III10	female		-	56, 59, 71	Yes/No
**Controls**	III3	female	56	-	-	No
	IV3	female	53	-	-	No

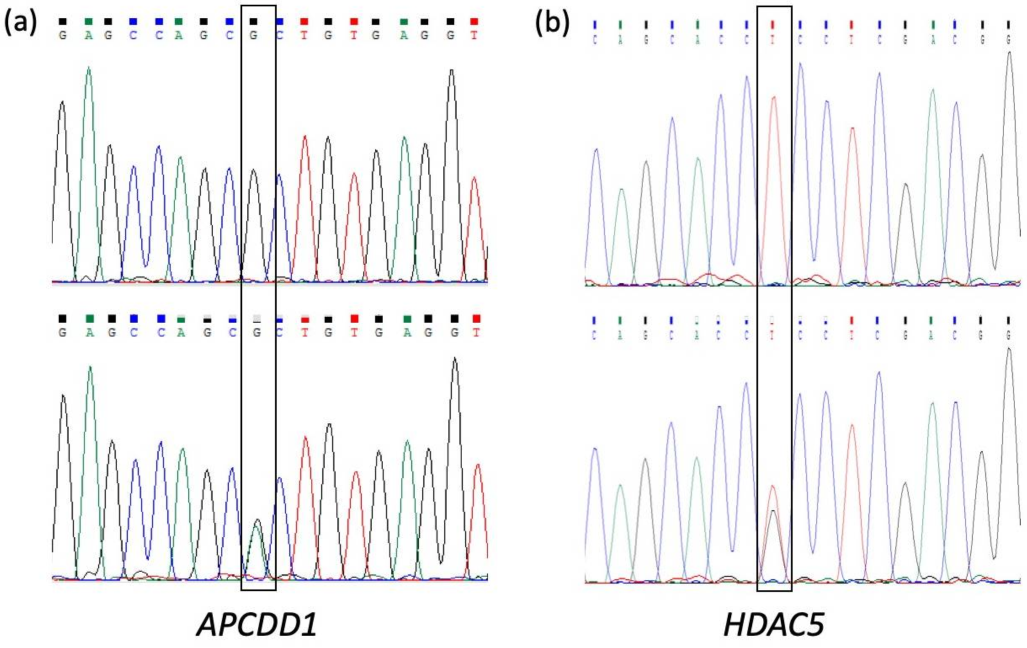

Figure S1. Representative electropherograms depicting the *APCDD1* and *HDAC5* variants identified in the studied CRC family. **(a)** Sanger sequencing confirmed the wild type sequence of *APCDD1* gene for family members II7, III3 and IV3 (upper panel) and the heterozygous missense variant (p.R299H) for the family members III7, III8 and III10. **(b)** Sanger sequencing confirmed the wild type sequence of *HDAC5* gene for family members III3, III10 and IV3 (upper panel) and the heterozygous 5′UTR variant (T → G) for family members II7, III7 and III8 (lower panel).

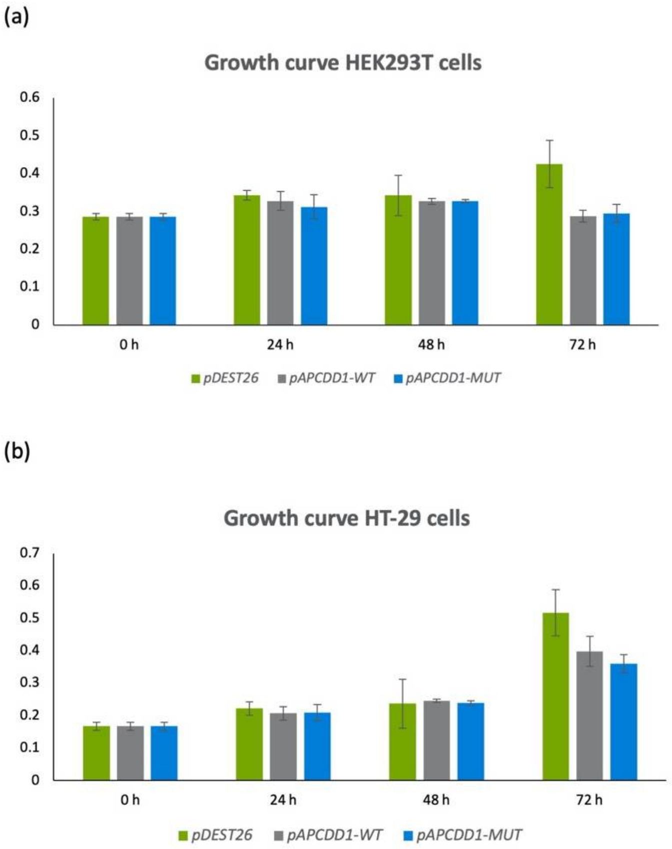

Figure S2. Cell proliferation assays conducted for *pAPCDD1*^{WT} and *pAPCDD1*^{MUT} using **(a)** HEK293T and **(b)** HT-29 cell lines. *pDEST26* vector was used as negative control. No significant increase of viable cell numbers was found between *pAPCDD1*^{WT} and *pAPCDD1*^{MUT} transfected cells at any measured time point ($p = 0.05$).

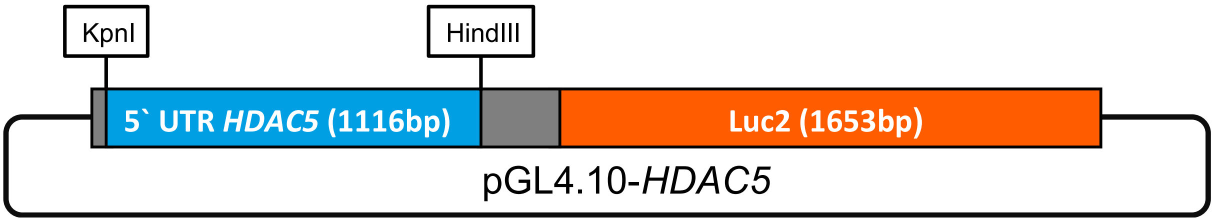

Figure S3. Graphical overview of pGL4.10-*HDAC5* reporter constructs. 5´UTR of *HDAC5* gene was cloned into the multiple cloning site directly upstream of the *luc2* reporter gene. Restriction sites for Kpn I and Hind III are indicated by arrows.
